# Supplementary material for: Rethinking remdesivir for COVID-19: A Bayesian reanalysis of trial findings
Source: PLoS One. 2021 Jul 23;16(7):e0255093. doi: 10.1371/journal.pone.0255093 (PMC8301659; doi:10.1371/journal.pone.0255093)
Supplement: S1 File — (PDF) [file pone.0255093.s001.pdf]

Rethinking Remdesivir for COVID-19: A Bayesian Reanalysis of Trial Results

Hoek, J.M., Field, S.M. de Vries, Y.A., Linde, M., Pittelkow, M.-M., Muradchanian, J., & van Ravenzwaaij, D.

March 22, 2021

Bayesian reanalyses of remdesivir trials for COVID-19

Bayes factors

At present, efficacy of a drug is most commonly determined employing *Null-Hypothesis Significance Testing* (NHST). Under NHST, a p-value is used to quantify the probability of obtaining the observed data or more extreme data under the assumption that the null hypothesis is true (typically, $\mathcal{H}_0$: no treatment effect). Therefore, the p-value for a two-sided test is $p = \Pr(X \geq |x| \mid \mathcal{H}_0)$. Using NHST, only evidence against but not in favour of the null hypothesis can be quantified. Bayes factors (BFs) offer a practical alternative to p-values and have many advantages over NHST. The most important one for the purpose of this manuscript is that Bayes factors allow contrasting the evidence in favour of the null and the alternative hypotheses simultaneously.

The Bayes factor is one form of Bayesian inference that is used for model comparison and hypothesis testing. Before considering any data, both the null hypothesis (e.g., no treatment effect) and the alternative hypothesis (e.g., treatment effect) are believed to have a certain probability of being true. These probabilities reflect our beliefs prior to seeing any data. Subsequently, the data are incorporated to update the prior probabilities into posterior probabilities (i.e., the probabilities of the hypotheses after having seen the data). The BF_{01} is the relative likelihood of the data under $\mathcal{H}_0$ compared to $\mathcal{H}_1$:

$$\underbrace{\frac{\Pr(\mathcal{H}_0 \mid D)}{\Pr(\mathcal{H}_1 \mid D)}}_{\text{Posterior odds}} = \underbrace{\frac{\Pr(D \mid \mathcal{H}_0)}{\Pr(D \mid \mathcal{H}_1)}}_{\text{Bayes factor, } \mathrm{BF}_{01}} \underbrace{\frac{\Pr(\mathcal{H}_0)}{\Pr(\mathcal{H}_1)}}_{\text{Prior odds}}$$

For example, if the Bayes factor is $\mathrm{BF}_{01} = 10$, the prior odds need to be updated by a factor of 10 to obtain the posterior odds; this can be interpreted as the data being 10 times more likely to have occurred under the null hypothesis compared to the alternative hypothesis. In other words, the data is 10 times more likely assuming that the treatment at hand is *not* effective compared to assuming that it is. Because of the advantages and the ease of interpretation of Bayes factors, we will use them in our reanalyses.

For our reanalyses we will use the packages BayesFactor (Morey & Rouder, 2018) and baymedr (Linde & van Ravenzwaaij, 2019), written in R (R Core Team, 2020).

```r
library("BayesFactor"); library("baymedr")
```

1A. Beigel et al. (2020) NEJM - Preliminary Efficacy Data From the NIAID ACTT-1 Study

The active drug is Remdesivir. The source of the presented information is: Beigel JH, Tomashek KM, Dodd LE, Mehta AK, Zingman BS, ... and Lane HC. Remdesivir for the treatment of Covid-19 – Preliminary report. New England Journal of Medicine. 2020 May 22.

The primary outcome

The primary clinical endpoint was time to clinical recovery, defined as the first day, during the 28 days after randomisation, on which a patient satisfied categories 1, 2 or 3 on an 8-category ordinal scale.

We have the following information:

> Patients in the remdesivir group had a shorter time to recovery than patients in the placebo group (median, 11 days, as compared with 15 days; rate ratio for recovery, 1.32; 95% confidence interval [CI], 1.12 to 1.55; $P < 0.001$; 1059 patients (Fig. 2 and Table 2).

Because we do not have access to the full data set or a published paper, we have to proceed with our analysis based on some assumptions. We present two alternative analysis strategies and show that they lead to virtually identical conclusions.

Strategy I: The t-test

The first analysis strategy treats the design as an independent two-sample t-test. We do not have access to within-group standard deviations or standard errors. We also assume that the data is normally distributed, which is questionnable as data in survival analysis is typically right-skewed. Based on the reported $n = 1059$ and $p < 0.001$, we can calculate a lower bound for the test statistic:

```r
Nplac = 521
Nrem = 538
Zrem = round (qt (.999, df = Nplac+Nrem-2), 2)
```

The test statistic 3.1 can subsequently be turned into a BF_{01} (Bayes factor for null over alternative). We will do a Bayesian two-sided t-test. For this and subsequent Bayesian t-tests, we calculate a Jeffreys-Zellner-Siow default Bayes factor. Under the alternative hypothesis, the prior distribution for the standardized effect size parameter δ is a Cauchy distribution with center zero and scale $1\sqrt(2)$ and the prior for σ^2 is $pr(\sigma^2) \propto 1/\sigma^2$. The analyses were conducted using the R package BayesFactor (Morey et al, 2018):

```r
BF10NIAID <- 1 / unname (ttest.tstat (
  t = Zrem,
  n1 = Nrem, n2 = Nplac,
  rscale = 1/sqrt(2),
  simple = TRUE
))
```

$\mathrm{BF}_{01} = 0.13$, meaning that the data is over 7 times more likely under a two-sided alternative hypothesis than it is under the null hypothesis. A sensitivity analysis with Cauchy prior scales for δ in the interval $[0.3, 1.5]$ yields a minimum BF_{01} of 0.07 at a Cauchy scale of 0.30 and a maximum BF_{01} of 0.26 at a Cauchy scale of 1.50.

Strategy II: The survival analysis

The second analysis strategy treats the design as a survival analysis. The test statistic is given by

$$Z = \frac{log(HR)}{SE(logHR)},$$

and $SE(logHR) = \frac{(log(CI+) - log(CI-))/2}{1.96}$, where $CI+$ and $CI-$ represent the upper and lower bounds of the 95% confidence interval, respectively. Based on this, we can calculate the test statistic:

```
Upper = 1.55; Lower = 1.12
SEHR = (log(Upper)-log(Lower)) / 2 / qnorm(.975)
HR = exp(mean(c(log(Upper), log(Lower))))
round (log(HR) / SEHR, 2)
```

```
## [1] 3.33
```

which is similar to the test statistic obtained under the first analysis strategy and should, for reasonable priors, lead to a similar Bayes factor.

The secondary outcome

The secondary clinical endpoint as reported was mortality rate at day 14. Mortality rates are reported as follows (Table 2):

- Remdesivir: 32 out of 538
- Placebo: 54 out of 521

With this information in hand, we calculate a BF_{01} (Bayes factor for null over alternative) for chi square tests. For this and subsequent Bayesian chi square tests, we test the null hypothesis of independent rows/columns against the alternative hypothesis of dependent rows/columns. Under the null hypothesis, the joint mortality rate parameter is $B(1, 1)$ distributed. Under the alternative hypothesis, independent mortality rate parameters for each group are both $B(1, 1)$ distributed. The analyses were conducted using the R package BayesFactor (Morey et al, 2018):

```
BF01chisq = round (1 / unname (as.vector (contingencyTableBF (
  x = matrix (
    data = c(32, 538-32, 54, 521-54),
    nrow = 2,
    ncol = 2
  ),
  sampleType = "indepMulti",
  fixedMargin = "cols"
))), 2)
```

$BF_{01} = 0.75$, meaning that the data is about equally likely under the null hypothesis as it is under the alternative.

Conclusion

The primary outcome analysis provides modest evidence to support the alternative hypothesis that Remdesivir outperforms a placebo, but the secondary outcome analysis provides ambiguous evidence about the efficacy of Remdesivir compared to a placebo. Overall, the results of this study can be classified as ambiguous.

1B. Beigel et al. (2020) NEJM - Final Efficacy Data From the NIAID ACTT-1 Study

The active drug is Remdesivir. The source of the presented information is: Beigel JH, Tomashek KM, Dodd LE, Mehta AK, Zingman BS, ... and Lane HC. Remdesivir for the treatment of Covid-19 – Final report. New England Journal of Medicine. 2020 November 5.

The primary outcome

The primary clinical endpoint was time to clinical recovery, defined as the first day, during the 28 days after randomisation, on which a patient satisfied categories 1, 2 or 3 on an 8-category ordinal scale.

We have the following information:

> Patients in the remdesivir group had a shorter time to recovery than patients in the placebo group (median, 10 days, as compared with 15 days; rate ratio for recovery, 1.29; 95% confidence interval [CI], 1.12 to 1.49; $P < 0.001$) (Fig. 2 and Table 2).

Because we do not have access to the full data set or a published paper, we have to proceed with our analysis based on some assumptions. We present two alternative analysis strategies and show that they lead to virtually identical conclusions.

Strategy I: The t-test

The first analysis strategy treats the design as an independent two-sample t-test. We do not have access to within-group standard deviations or standard errors. We also assume that the data is normally distributed, which is questionnable as data in survival analysis is typically right-skewed. Based on the reported $n = 1062$ and $p < 0.001$, we can calculate a lower bound for the test statistic:

```
Nplac = 521
Nrem = 541
Zrem = round (qt (.999, df = Nplac+Nrem-2), 2)
```

The test statistic 3.1 can subsequently be turned into a BF_{01} (Bayes factor for null over alternative). We will do a Bayesian two-sided t-test:

```
BF10NIAID <- 1 / unname (ttest.tstat (
  t = Zrem,
  n1 = Nrem, n2 = Nplac,
  rscale = 1/sqrt(2),
  simple = TRUE
))
```

$\text{BF}_{01} = 0.13$, meaning that the data is over 7 times more likely under a two-sided alternative hypothesis than it is under the null hypothesis. A sensitivity analysis with Cauchy prior scales for δ in the interval $[0.3, 1.5]$ yields a minimum BF_{01} of 0.07 at a Cauchy scale of 0.30 and a maximum BF_{01} of 0.26 at a Cauchy scale of 1.50.

Strategy II: The survival analysis

The second analysis strategy treats the design as a survival analysis. The test statistic is given by

$$Z = \frac{log(HR)}{SE(logHR)},$$

and $SE(logHR) = \frac{(log(CI+) - log(CI-))/2}{1.96}$, where $CI+$ and $CI-$ represent the upper and lower bounds of the 95% confidence interval, respectively. Based on this, we can calculate the test statistic:

```
Upper = 1.49; Lower = 1.12
SEHR = (log(Upper)-log(Lower)) / 2 / qnorm(.975)
HR = exp(mean(c(log(Upper), log(Lower))))
round (log(HR) / SEHR, 2)
```

```
## [1] 3.52
```

which is larger than the test statistic obtained under the first analysis strategy and should, for reasonable priors, lead to a larger Bayes factor.

The secondary outcome

The secondary clinical endpoint as reported was mortality rate at days 15 and 29. Mortality rates are reported as follows (Table 2):

- Remdesivir (day 15): 35 out of 541
- Placebo (day 15): 61 out of 521
- Remdesivir (day 29): 59 out of 541
- Placebo (day 29): 77 out of 521

With this information in hand, we calculate two BF_{01}s (Bayes factor for null over alternative). We will do two Bayesian chi square tests:

```
BF01chisq15 = round (1 / unname (as.vector (contingencyTableBF (
  x = matrix (
    data = c(35, 541-35, 61, 521-61),
    nrow = 2,
    ncol = 2
  ),
  sampleType = "indepMulti",
  fixedMargin = "cols"
))), 2)

BF01chisq29 = round (1 / unname (as.vector (contingencyTableBF (
  x = matrix (
    data = c(59, 541-59, 77, 521-77),
    nrow = 2,
    ncol = 2
  ),
  sampleType = "indepMulti",
  fixedMargin = "cols"
))), 2)
```

$BF_{01}s =$ of 0.27 (the data provides moderate evidence for the alternative hypothesis) and 3.29 (the data provides moderate evidence for the null hypothesis) for 15 and 29 day mortality, respectively.

Conclusion

The primary outcome analysis provides modest evidence to support the alternative hypothesis that Remdesivir outperforms a placebo, but the secondary outcome analysis provides ambiguous evidence about the efficacy of Remdesivir compared to a placebo. Overall, the results of this study can be classified as ambiguous.

2. Goldman et al. (2020) NEJM - Efficacy Data From Gilead's SIMPLE Trial (Study GS-US-540-5773)

The active drug is Remdesivir, compared were a 5-day regimen to a 10-day regimen. The source of the presented information is:

Goldman JD, Lye DC, Hui DS, Marks KM, Bruno R, . . . and, Subramanian A. Remdesivir for 5 or 10 days in patients with severe Covid-19. New England Journal of Medicine. 2020 May 27.

The primary outcome

The primary clinical endpoint was clinical improvement at 14 days after randomisation. Clinical improvement was defined as a two-point reduction in patients' admission status on a seven-point ordinal scale.

Clinical improvement rates are reported as follows (Table 2):

- 5-day regimen: 129 out of 200
- 10-day regimen: 107 out of 197

Note that in this trial, the proportion of patients that show improvement is comparatively large. With this information in hand, we calculate a BF_{01} (Bayes factor for null over alternative). We will do a Bayesian chi square test, with the null hypothesis being independent rows/columns:

```
BF01chisq = round (1 / unname (as.vector (contingencyTableBF (
  x = matrix (
    data = c(129, 200-129, 107, 197-107),
    nrow = 2,
    ncol = 2
  ),
  sampleType = "indepMulti",
  fixedMargin = "cols"
))), 2)
```

$BF_{01} = 0.97$, meaning that the data is about as likely under the alternative hypothesis as it is under the null hypothesis.

The secondary outcome

One of the secondary clinical endpoints was mortality rate at day 14. Mortality rates are reported as follows (Table 2):

- 5-day regimen: 16 out of 200
- 10-day regimen: 21 out of 197

With this information in hand, we can calculate a BF_{01} (Bayes factor for null over alternative). We do a Bayesian chi square test:

```
BF01chisq = round (1 / unname (as.vector (contingencyTableBF (
  x = matrix (
    data = c(16, 200-16, 21, 197-21),
    nrow = 2,
    ncol = 2
  ),
  sampleType = "indepMulti",
  fixedMargin = "cols"
))), 2)
```

$BF_{01} = 9.1$, meaning that the data is over 9 times more likely under the null hypothesis than it is under the alternative.

Conclusion

The secondary outcome analysis provides modest support in favour of the null hypothesis that 5-day treatment is equally effective as 10-day treatment.

3. Wang et al. (2020), The Lancet

The active drug is Remdesivir. The source of the presented information is:
Wang Y, Zhang D, Du G, Du R, Zhao J, ... and Wang C. Remdesivir in adults with severe COVID-19: a randomised, double-blind, placebo-controlled, multicentre trial. The Lancet. 2020 Apr 29.

The primary outcome, full group

The primary clinical endpoint was time to clinical improvement within 28 days after randomisation. Clinical improvement was defined as a two-point reduction in patients' admission status on a six-point ordinal scale, or live discharge from the hospital, whichever came first.

Strategy I: The *t*-test

The first analysis strategy treats the design as an independent two-sample *t*-test. Because we do not have raw data, we have to analyse using reported descriptive statistics. We have the following information (Table 3):

```
nRem = 158;           nPlac = 78
medianRem = 21;       medianPlac = 23
IQRRem = c(13, 28);   IQRPlac = c(15, 28)
```

	n	First quartile	Median	Third quartile
Remdesivir	158	13	21	28
Placebo	78	15	23	28

This means we do not have access to the standard deviations. Furthermore, data is unlikely to be normally distributed, as the maximum value of 28 is the upper range of both IQRs. Fortunately, *t*-tests are fairly robust against non-normality. We will use the IQRs combined with the assumption of approximate normality to obtain an educated guess for the standard deviations:

```
IQRtoSD = qnorm(.75) * 2 # IQR to sd ratio if distribution were standard normal.
sdRem = (IQRRem[2]-IQRRem[1]) / IQRtoSD
sdPlac = (IQRPlac[2]-IQRPlac[1]) / IQRtoSD
```

With this information in hand, we can calculate a BF_{01} (Bayes factor for null over alternative). The analyses were conducted using the R package baymedr (Linde & van Ravenzwaaij, 2019):

```
1 / get_bf(super_bf(
  n_x = nPlac, n_y = nRem,
  mean_x = medianPlac, mean_y = medianRem,
  sd_x = sdPlac, sd_y = sdRem,
  direction = "low",
  alternative = "two.sided"
))
```

```
## [1] 2.802276
```

$BF_{01} = 2.80$, meaning that the data is 2.80 times more likely under the null hypothesis than it is under a two-sided alternative. A sensitivity analysis with Cauchy prior scales for δ in the interval $[0.3, 1.5]$ yields a minimum BF_{01} of 1.54 at a Cauchy scale of 0.30 and a maximum BF_{01} of 5.54 at a Cauchy scale of 1.50.

Strategy II: The survival analysis

The second analysis strategy treats the design as a survival analysis. The test statistic is given by

$$Z = \frac{log(HR)}{SE(logHR)},$$

and $SE(logHR) = \frac{(log(CI+)-log(CI-))/2}{1.96}$, where $CI+$ and $CI-$ represent the upper and lower bounds of the 95% confidence interval, respectively. Based on this, we can calculate the test statistic:

```
Upper = 1.75; Lower = 0.87
SEHR = (log(Upper)-log(Lower))/2/qnorm(.975)
HR = exp(mean(c(log(Upper), log(Lower))))
round (log(HR) / SEHR, 2)
```

```
## [1] 1.18
```

which is similar to the test statistic obtained under the first analysis strategy (1.356497) and should, for reasonable priors, lead to a similar Bayes factor.

The primary outcome, early treatment group

This subgroup analysis examines time to clinical improvement in the subgroup of people who received Remdesivir within 10 days of symptom onset. We have the following information (page 5):

> Although not statistically significant, in patients receiving remdesivir or placebo within 10 days of symptom onset in the ITT population, those receiving remdesivir had a numerically faster time to clinical improvement than those receiving placebo (median 18 · 0 days [IQR 12 · 0–28 · 0] vs 23 · 0 days [15 · 0–28 · 0]; HR 1 · 52 [0 · 95–2 · 43]; appendix p 6).

Strategy I: The t-test

The first analysis strategy treats the design as an independent two sample t-test. Because we do not have raw data, we have to analyse using reported descriptive statistics from the quote above and from Table 3 (sample sizes):

```
nRem = 71;              nPlac = 47
medianRem = 18;         medianPlac = 23
IQRRem = c(12, 28);    IQRPlac = c(15, 28)
```

	n	First quartile	Median	Third quartile
Remdesivir	71	12	18	28
Placebo	47	15	23	28

This means we do not have access to the standard deviations. Furthermore, data is unlikely to be normally distributed, as the maximum value of 28 is the upper range of both IQRs. Fortunately, t-tests are fairly robust against non-normality. We will use the IQRs combined with the assumption of approximate normality to get an educated guess for the standard deviations:

```
IQRtoSD = qnorm(.75) * 2 # IQR to sd ratio if distribution were standard normal.
sdRem = (IQRRem[2]-IQRRem[1]) / IQRtoSD
sdPlac = (IQRPlac[2]-IQRPlac[1]) / IQRtoSD
```

With this information in hand, we can calculate a BF_{01} (Bayes factor for null over alternative). The analyses were conducted using the R package baymedr (Linde & van Ravenzwaaij, 2019):

```
1 / get_bf(super_bf(
  n_x = nPlac, n_y = nRem,
  mean_x = medianPlac, mean_y = medianRem,
  sd_x = sdPlac, sd_y = sdRem,
  direction = "low",
  alternative = "two.sided"
))
```

```
## [1] 0.3814308
```

$BF_{01} = 0.38$, meaning that the data is $1/0.38 = 2.62$ times more likely under a two-sided *alternative* hypothesis than it is under the null hypothesis. A sensitivity analysis with Cauchy prior scales for δ in the interval $[0.3, 1.5]$ yields a minimum BF_{01} of 0.32 at a Cauchy scale of 0.32 and a maximum BF_{01} of 0.63 at a Cauchy scale of 1.50.

Strategy II: The survival analysis

The second analysis strategy treats the design as a survival analysis. The test statistic is given by

$$Z = \frac{log(HR)}{SE(logHR)},$$

and $SE(logHR) = \frac{(log(CI+)-log(CI-))/2}{1.96}$, where $CI+$ and $CI-$ represent the upper and lower bounds of the 95% confidence interval, respectively. Based on this, we can calculate the test statistic:

```
Upper = 2.43; Lower = 0.95
SEHR = (log(Upper)-log(Lower))/2/qnorm(.975)
HR = exp(mean(c(log(Upper), log(Lower))))
log(HR) / SEHR
```

```
## [1] 1.745878
```

which is smaller than the test statistic obtained under the first analysis strategy (2.410047) and should, for reasonable priors, lead to a Bayes factor relatively more in favor of the null hypothesis.

The secondary outcome

One of the secondary clinical endpoints was mortality rate at day 28.

Full group

Mortality rates are reported as follows (Table 3):

- Remdesivir: 22 out of 158
- Placebo: 10 out of 78

With this information in hand, we can calculate a BF_{01} (Bayes factor for null over alternative). We will do a Bayesian chi square test:

```
BF01chisq = round (1 / unname (as.vector (contingencyTableBF (
  x = matrix(
    data = c(22, 158-22, 10, 78-10),
    nrow = 2,
    ncol = 2
  ),
  sampleType = "indepMulti",
```

```
  fixedMargin = "cols"
))), 2)
```

$BF_{01} = 8.34$, meaning that the data is over 8 times more likely under the null hypothesis than it is under the alternative.

Early treatment group

Mortality rates for the subgroup of people who received Remdesivir within 10 days of symptom onset are reported as follows (Table 3):

- Remdesivir: 8 out of 71
- Placebo: 7 out of 47

With this information in hand, we can calculate a BF_{01} (Bayes factor for null over alternative). We will do a Bayesian chi square test:

```
BF01chisq = round (1 / unname (as.vector (contingencyTableBF (
  x = matrix (
    data = c(8, 71-8, 7, 47-7),
    nrow = 2,
    ncol = 2
  ),
  sampleType = "indepMulti",
  fixedMargin = "cols"
))), 2)
```

$BF_{01} = 5.37$, meaning that the data is over 5 times more likely under the null hypothesis than it is under the alternative.

Conclusion

These efficacy analyses provide modest evidence to support the null hypothesis, one provides weak evidence to support the alternative hypothesis. Although the evidence is not strong, it would be unwise to ignore this study altogether for purposes of making endorsement decisions.

4. WHO Solidarity Trial Consortium (2021), NEJM

The active drug is Remdesivir. The source of the presented information is:
WHO Solidarity Trial Consortium. Repurposed Antiviral Drugs for Covid-19 — Interim WHO Solidarity Trial Results. The New England Journal of Medicine. 2021 Feb 11.

The primary outcome

The primary clinical endpoints was in-hospital mortality rate.

Mortality rates are reported as follows (Figure 3):

- Remdesivir: 301 out of 2743
- Placebo: 303 out of 2708

With this information in hand, we can calculate a BF_{01} (Bayes factor for null over alternative). We will do a Bayesian chi square test:

```
BF01chisq = round (1 / unname (as.vector (contingencyTableBF (
  x = matrix (
    data = c(301, 2743-301, 303, 2708-303),
    nrow = 2,
```

```
    ncol = 2
  ),
  sampleType = "indepMulti",
  fixedMargin = "cols"
))), 2)
```

$BF_{01} = 45.44$, meaning that the data is over 45 times more likely under the null hypothesis than it is under the alternative.

The secondary outcome

One of the two secondary outcomes was the initiation of mechanical ventilation. Mortality rates for the group without mechanical ventilation are reported as follows (Figure 3):

- Remdesivir: 203 out of 2489
- Placebo: 232 out of 2475

With this information in hand, we can calculate a BF_{01} (Bayes factor for null over alternative). We will do a Bayesian chi square test:

```
BF01chisq = round (1 / unname (as.vector (contingencyTableBF (
  x = matrix (
    data = c(203, 2489-203, 232, 2475-232),
    nrow = 2,
    ncol = 2
  ),
  sampleType = "indepMulti",
  fixedMargin = "cols"
))), 2)
```

$BF_{01} = 15.75$, meaning that the data is over 15 times more likely under the null hypothesis than it is under the alternative.

5. Spinner et al. (2020), JAMA

The active drug is Remdesivir, compared were a 5-day regimen to a 10-day regimen, and a placebo group was included. The source of the presented information is:

Spinner, CD, Gottlieb, RL, Criner, GJ, Lopez, JRA; Cattelan, AM, … and Marty, FM. Effect of Remdesivir vs Standard Care on Clinical Status at 11 Days in Patients With Moderate COVID-19: A Randomized Clinical Trial. Journal of the American Medical Association. 2020 Aug 21.

The primary outcome

The primary efficacy end point was the distribution of clinical status assessed on a 7-point ordinal scale on study day 11. The scale ranged from "1. Death" to "7. Not hospitalized". Frequencies of scores 1 through 7 in each of the three groups were reported as follows (Table 2):

- Remdesivir-5: 0, 0, 5, 7, 38, 7, 134
- Remdesivir-10: 2, 1, 0, 12, 44, 9, 125
- Placebo: 4, 4, 7, 11, 46, 8, 120

The analysis strategy in the target article used a non-parametric approach, but we believe a parametric approach is appropriate (and thus preferable) if scores 1 through 6 are grouped together (hospitalized/death).

With this information in hand, we can calculate a BF_{01} (Bayes factor for null over alternative). We will do a Bayesian chi square test:

```r
BF01chisq5 = round (1 / unname (as.vector (contingencyTableBF (
  x = matrix (
    data = c(57, 134, 80, 120),
    nrow = 2,
    ncol = 2
  ),
  sampleType = "indepMulti",
  fixedMargin = "cols"
))), 2)

BF01chisq10 = round (1 / unname (as.vector (contingencyTableBF (
  x = matrix (
    data = c(68, 125, 80, 120),
    nrow = 2,
    ncol = 2
  ),
  sampleType = "indepMulti",
  fixedMargin = "cols"
))), 2)
```

$\text{BF}_{01}s =$ of 0.91 and 5.12 for respectively Remdesivir-5 and Remdesivir-10 compared against placebo. This means that depending on the group, the data are either equally likely under either hypothesis or slightly more likely under the null hypothesis than under the alternative.

References

- FDA. Fact sheet for health care providers - Emergency use authorization (EUA) of remdesivir (GS-5734tm) [Internet]. FDA; 2020 May 1. Available from: https://www.fda.gov/media/137566/download
- Gilead Sciences. Gilead Announces Results From Phase 3 Trial of Investigational Antiviral Remdesivir in Patients With Severe COVID-19 [Internet]. Gilead Sciences. 2020 Apr 29. Available from: http://investors.gilead.com/news-releases/news-release-details/gilead-announces-results-phase-3-trial-investigational-antiviral
- Linde M, van Ravenzwaaij D. baymedr: Computation of Bayes factors for common biomedical designs, 2019. URL https://CRAN.R-project.org/package=baymedr. R package version 0.1.0.
- Morey R D, Rouder J N. BayesFactor: Computation of Bayes factors for common designs, 2018. URL https://CRAN.R-project.org/package=BayesFactor. R package version 0.9.12-4.2.
- NIAID Office of Communications. NIH Clinical Trial Shows Remdesivir Accelerates Recovery from Advanced COVID-19 | NIH: National Institute of Allergy and Infectious Diseases [Internet]. 2020 Apr 29. Available from: http://www.niaid.nih.gov/news-events/nih-clinical-trial-shows-remdesivir-accelerates-recovery-advanced-covid-19
- R Core Team. R: A language and environment for statistical computing. R Foundation for Statistical Computing, Vienna, Austria, 2020. URL https://www.R-project.org/.
- Wang Y, Zhang D, Du G, Du R, Zhao J, Jin Y, Fu S, Gao L, Cheng Z, Lu Q, Hu Y. Remdesivir in adults with severe COVID-19: a randomised, double-blind, placebo-controlled, multicentre trial. The Lancet. 2020 Apr 29.
