# Supplementary material for: Rethinking remdesivir for COVID-19: A Bayesian reanalysis of trial findings
Source: PLoS One. 2021 Jul 23;16(7):e0255093. doi: 10.1371/journal.pone.0255093 (PMC8301659; doi:10.1371/journal.pone.0255093)
Supplement: S1 Table — (PDF) [file pone.0255093.s003.pdf]

S1 Table. Hoek, Field et al. (2021)

Table A: Overview of Bayes factors of reanalyzed studies at time of initial regulatory approvals

Study	Comparators	Subgroup	Outcome	BF_{01}	Evidential Strength (14)
ACTT-1 Preliminary report Beigel et al (2020)	10-day remdesivir vs. placebo	Full patient group	Time to clinical recovery in days	0.13	Moderate; pro-remdesivir
			Mortality rate at day 14	0.75	Ambiguous
Wang et al. (2020)	10-day remdesivir vs. placebo	Full patient group	Time to clinical improvement in days	2.80	Ambiguous
			Mortality rate at day 28	8.34	Moderate; pro no effect
		Treatment within 10 days of symptom onset	Time to clinical improvement in days	0.38	Ambiguous
			Mortality rate at day 28	5.37	Moderate; pro no effect
GS-US-540-5773 (Goldman et al., 2020)	5- vs. 10-day remdesivir treatment	-	Clinical improvement rate at day 14	0.97	Ambiguous
			Mortality rate at day 14	9.10	Moderate; pro equal effect 5- vs. 10- day treatment

Table B: Overview of Bayes factors of reanalyzed studies that became available after initial regulatory approval of remdesivir.

ACTT-1<br>Final report<br>(Beigel et al., 2020)	10-day remdesivir<br>vs. placebo	Full patient group	Time to clinical recovery in days	0.13	Moderate; pro-remdesivir
			Mortality rate at day 15	0.27	Moderate; pro-remdesivir
			Mortality rate at day 29	3.29	Moderate; pro no treatment effect
Spinner et al., (2020)	5-day remdesivir,<br>vs. standard of care	-	Clinical improvement rate at day 11	0.91	Ambiguous
	10-day remdesivir,<br>vs. standard of care	-	Clinical improvement rate at day 11	5.12	Moderate; pro no treatment effect
WHO Solidarity		Full patient group	In hospital mortality rate	45.44	Strong; pro no treatment effect
		Patients without mechanical ventilation	In hospital mortality rate	15.75	Strong; pro no treatment effect
