# Supplementary material for: Rethinking remdesivir for COVID-19: A Bayesian reanalysis of trial findings
Source: PLoS One. 2021 Jul 23;16(7):e0255093. doi: 10.1371/journal.pone.0255093 (PMC8301659; doi:10.1371/journal.pone.0255093)
Supplement: Attachment — Submitted filename: Response to Reviews Hoek, Field et al.docx [file pone.0255093.s004.docx]

Dear sir, madam,

Below, we include the response to reviews of our manuscript “Rethinking remdesivir for COVID-19: a Bayesian reanalysis of trial findings” (PONE-D-20-30134) for publication in *PLOS ONE*. We start our response by pointing out the changes that we made to the manuscript since its original submission. In addition, we reproduced the relevant sections of the reviews in standard font and added our response in bold. We look forward to your comments.

Kind regards,

Joyce Hoek and Sarahanne Field

# Changes made to the manuscript since first submission.

**In our manuscript, we reanalyze the available clinical data of remdesivir using Bayesian methods to provide a more complete picture of the current state of evidence. Since we first submitted our manuscript in October 2020, new clinical trial data assessing the efficacy of remdesivir has been published. We find it important to include this new information in our manuscript as well. This provides a more complete and accurate picture of the current state of evidence for the efficacy of remdesivir against COVID-19. Nevertheless, the focus of our paper did not change. The three studies that were used as the basis for marketing authorization of remdesivir remain the focus of our paper.**

**We included an explanation of the new studies on lines 18-22 of page 3, lines 10-15 of page 4, lines 23-25 of page 8, lines 1-5 of page 9, and the section “Reanalysis of studies published after initial marketing approval of remdesivir” of our Results.**

# Response to reviews.

Reviewer #1

R1-1 The authors should give explanation why a Bayesian method is preferred than a frequentist one for this problem?

**In our opinion, there are many advantages to Bayesian hypothesis testing over frequentist hypothesis testing, such as the ability to continually test as data comes in (sequential testing) and quantifying the likelihood of hypotheses relative to one another. In the context of this specific reanalysis, the main advantage is the ability to quantify evidence in favor of the null hypothesis (i.e., the absence of efficacy), which traditional p-values do not allow for.**

**When medicine regulators initially approved remdesivir, limited clinical trial data was available. In addition, one of the two available randomized placebo-controlled trials by Wang et al. could not be interpreted in a frequentist framework. Since we think it is important to include both trials in the regulatory efficacy assessment, we present a Bayesian reanalysis of the data.**

**We agree that the original manuscript did not state explicitly why a Bayesian method is preferred in this specific case. We expanded our explanation of the added value of a Bayesian approach at lines 12-13, 19-25 of page 2 and lines 5-13 of page 3 of our manuscript.**

R1-2 The authors should try to provide the data links, so the data become public accessible. Or at least provide the summary statistics of the data as they used in this paper, either in the text or in an Appendix.

**All data and analysis code is available on the public OSF repository at this url:** [**https://osf.io/kdqt3**](https://osf.io/kdqt3)**. We now also included this link in our manuscript at line 24 of page 3.**

This will help the public researchers to evaluate performance of remdivir and the that of the existing studies on remdivir.

**With regard to this second part of your comment, we wanted to facilitate the interpretation of our results by replacing the table of our results with a figure. This figure provides an overview of the current state of evidence for remdesivir in the treatment of COVID-19. The figure is presented at page 7 of our manuscript. We moved the table to the supplementary material.**

R1-3 The authors mentioned that Jeffreys-Zellner-Siow Bayes factors based on Bayesian t-test and chi-squared test were used in the reanalysis. Please discuss the advantage of this method vs the classical Bayes factor.

**The Jeffreys-Zellner-Siow Bayes factor has as distinguishing property the use of the Cauchy prior, centered on zero. Citing from van Ravenzwaaij & Etz (in press, available at** [**https://psyarxiv.com/27ndb/**](https://psyarxiv.com/27ndb/)**):**

***"The Cauchy prior has some desirable mathematical properties (see e.g., Bayarri, Berger, Forte, & García-Donato, 2012; Consonni, Fouskakis, Liseo, & Ntzoufras, 2018), such as model selection consistency (for data generated under a model, the corresponding Bayes factor should go to infinity as sample size goes to infinity), predictive matching (a minimum sample size should exist for which the Bayes factor is 1, such that models are indistinguishable), and information consistency (a minimum sample size should exist for which data that result in test statistics that go to infinity should have corresponding Bayes factors that also go to infinity). Other priors may share some of these desirable properties, but the Cauchy prior has caught on as the go-to choice because it satisfies them all and is relatively easy to specify and interpret."***

**We included an explanation on lines 3-8 of page 5 of our manuscript.**

R1-4 The authors mentioned that non-informative priors are used in their analysis. As there are several different non-informative priors, please specify which non-informative prior is used.

**We now included a more extensive discussion of the methods that we used and our choice of priors under the subheadings ‘Bayesian two-sided *t*-test’ and ‘Bayesian chi-square test’ on page 4 and 5 of our manuscript and in our supplementary material.**

Reviewer #2

R2-1 This manuscript used Bayes factor to re-analyze the three trials on Remdesivir for treating COVID-19. The manuscript is well-written but has very limited contribution to either statistical method or clinical guidance. There is no discussion of why BF is used and how it improves the conclusions.

**As indicated in our response to R1-1, our main reason for conducting the Bayesian reanalyses is to be able to quantify pro-null evidence. Under a frequentist framework, it remains unclear whether non-significant results (such as those by Wang et al.) are due to lack of power or due to null effects. Perhaps more importantly, concerns about "perceived significance" should not be a reason for rejection at *PLOS ONE*, and the reviewer does not present any compelling methodological concerns that could motivate a rejection.**

**In addition, we disagree that our paper has limited contribution to clinical guidance. When remdesivir was first approved, only two relevant clinical trials were available. One of these trials could not be interpreted under a frequentist framework and was excluded from regulatory assessments of remdesivir. When evidence is limited, it is important that all available evidence can be assessed. We provide a method to do so.**

**As explained in our response to reviewer one, we did include a more explicit explanation of the added value of a Bayesian approach in this specific case in the introduction of our manuscript.**

R2-2 There is no discussion of using and how to use different prior information.

**We have added detailed information about the choice of priors for both the Bayesian t-test and the Bayesian chi-square test to both the manuscript (page 4 and 5) and in the supplementary material.**

R2-3 The authors can consider combining information from the three trials to improve conclusion.

**Thank you for your suggestion. We agree and are enthusiastic about combining information from the three trials, but as the data are quite different, we prefer to stick to combining the information qualitatively as we do in our current version of the manuscript. A (Bayesian) meta-analysis would not be justified, given the underlying assumptions. We explain our reasoning on lines 1-15 of page 4 of our manuscript.**
